# Supplementary material for: Directive giant upconversion by supercritical bound states in the continuum
Source: Nature. 2024 Feb 21;626(8000):765–71. doi: 10.1038/s41586-023-06967-9 (PMC10881401; doi:10.1038/s41586-023-06967-9)
Supplement: Supplementary file 1 — The Supplementary Information includes notes on further numerical simulations; an appendix on the FW quantum model; methods for NP synthesis and optical characterization; detailed fabrication; band diagrams and upconversion characterization set-ups; methods for enhancement factor estimation; sample size influence. The notes are accompanied by Supplementary Figs. 1–14. [file 41586_2023_6967_MOESM1_ESM.pdf]

nature portfolio

https://doi.org/10.1038/s41586-023-06967-9

Supplementary information

Directive giant upconversion by supercritical bound states in the continuum

In the format provided by the authors and unedited

Supplementary Materials for

Directive Giant Upconversion by Supercritical Bound States in the Continuum

Chiara Schiattarella, [1] Silvia Romano, [1] Luigi Sirleto [1], Vito Mocella, [1] Ivo Rendina, [2] Vittorino Lanzio, [3] Stefano Cabrini, [3] Fabrizio Riminucci,[3] Adam Schwartzberg,[3] Jiaye Chen,[4] Liangliang Liang, [4] Xiaogang Liu, [4,5,6*] Gianluigi Zito [1*]

[1]Institute of Applied Sciences and Intelligent Systems, National Research Council, Naples, Italy
[2]Institute of Applied Sciences and Intelligent Systems, National Research Council, Pozzuoli, Italy
[3]Molecular Foundry, Lawrence Berkeley National Laboratory, Berkeley, California, United States
[4]Department of Chemistry, National University of Singapore, 117543, Singapore.
[5]Institute of Materials Research and Engineering, Agency for Science, Technology and Research, Singapore, 138634, Singapore
[6]Center for Functional Materials, National University of Singapore Suzhou Research Institute, Suzhou 215123, China.

*To whom correspondence should be addressed; E-mail: chmlx@nus.edu.sg (X. Liu); gianluigi.zito@na.isasi.cnr.it. (G. Zito)

Contents

1.1 Numerical simulations (RCWA and FDTD) and related experimental data 3

1.2 Appendix: Friedrich-Wintgen quantum model 6

1.3 Quasi symmetry-protected BICs *versus* Friedrich-Wintgen quasi-BICs 10

2 Synthesis of Upconversion photoluminescence (UCPL) NPs 10

2.1 Materials 10

2.2 Synthesis of $NaErF_4$ core nanocrystals 10

2.3 Synthesis of $NaErF_4$@$NaYF_4$ core-shell nanocrystals 11

2.4 Synthesis of $NaGdF_4$:Nd/Yb(40/5%) @$NaGdF_4$:Yb/Tm(49/1%) @$NaGdF_4$:Eu(15%) core-shell-shell nanocrystals 11

3 Characterization of the as-produced NPs 11

4 Experimental samples 14

4.1 PCNS fabrication 14

4.2 Upconversion Nanoparticles (UCNPs) deposition and morphology 14

5 Dispersion band diagram measurements 16

6 UCPL measurements: Experimental interrogation and detection 20

7 Enhancement factor estimation 21

8 Additional FDTD simulations 26

9 UCPL emission scaling with pattern size 27

1.1 Numerical simulations (RCWA and FDTD) and related experimental data

Bloch periodic boundary conditions along *x*- and *y*-directions were used. On the top and bottom surfaces normal to the *z*-direction, perfectly-matched-layer (PML) absorbing boundary conditions were used. The adapted mesh along the *z*-direction had a size step of 3 nm inside the PCNS and was increased up to a value of 20 nm outside. PML was also used for x-y directions to evaluate the transmittance spectra in finite superstructures of hundreds of unit cells, as described below. The finite-different time-domain (FDTD) method was similarly applied in the next section to simulate the radiation pattern at the boundary of a finite PCNS. Additional finite element method-based simulations, performed with Comsol Multiphysics 6.0, were used to verify the numerical results. The dispersion used for simulations was measured (**Supplementary Fig. 1**) and fitted in Lumerical FDTD with fit tolerance 10^{-6}, max coefficients 6 and imaginary weight 100**,** leading to an imaginary part of the refractive index used for simulations equal to $n_I = 10^{-4}$ over the spectral range 700 – 1200 nm.

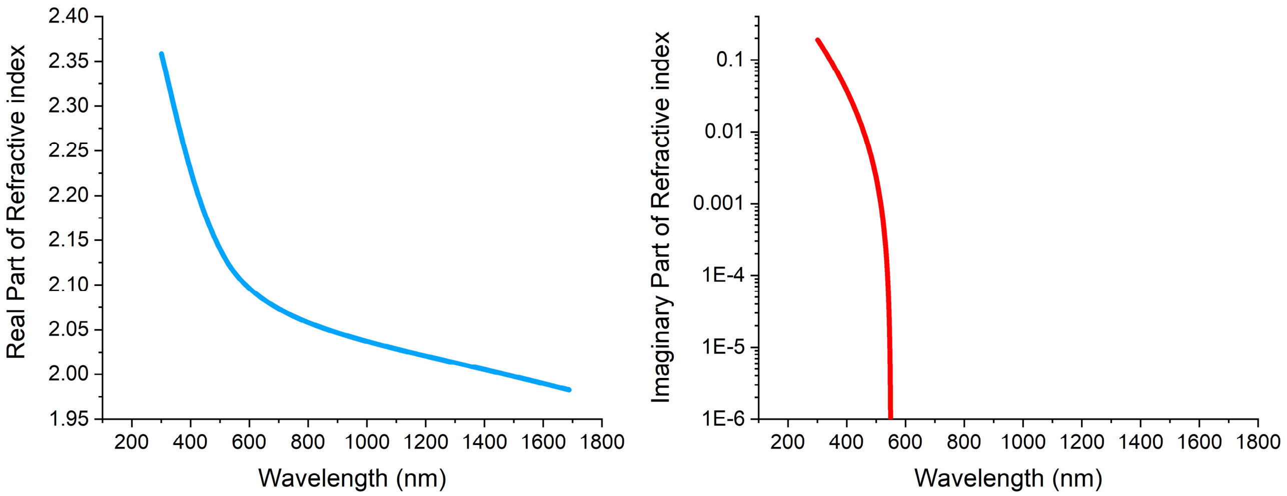

Supplementary Fig. 1 | Experimental complex refractive index of the silicon nitride film (UVISEL Plus, Horiba Jobin Yvon, Spectroscopic Ellipsometer), used for numerical simulations (Lumerical 2022 R1, RCWA module).

Supplementary Figure 2 shows the evolution of the interference process as a function of κ_{12} and describes how the coupling changes at the edge. Band-diagrams were calculated with RCWA in an infinite structure (top) and in a finite superstructure (bottom) at the boundary with a unform slab (finite structure of 200 X 200 unitary cells and equivalent size of unform slab). The reduction in separation of the two modes - their near field coupling - is also simulated by perturbing the effective material index. In this second formulation, it is considered indeed that at the boundary of the PCNS, the mode optical fields are evanescently overlapped with a region of different (slightly larger) average refractive index, which can be seen as a perturbation of the effective material index of the slab seen by the modes. The change in effective material index has the same effect as the change in proximity to the boundary. It disturbs the modal distribution, which affects its effective mode index and mutual coupling κ_{12}. Thus, changing the effective material index is a simple way to visualize and display the boundary effect, since it also changes mode fields and the integral overlap κ_{12}, reproducing the experimental result at the PCNS edge in **Fig. 3a**.

The effect of the finite boundary on resonance was investigated using near-field scanning optical microscopy (Witec Alpha RAS 300) with a scaled geometry sustaining BIC at 532 nm. This investigation was repeated over a set of samples with different geometries. For all of them, the mode was found to be perturbed at the boundary only on the last unitary cell, making the system robust at the edge as well. Recall that it was also found in [4] that the quasi-BIC is robust even with an 8 x 8 unit cell array. This scanning near-field optical microscopy (SNOM) map is shown in **Supplementary Fig. 3.**

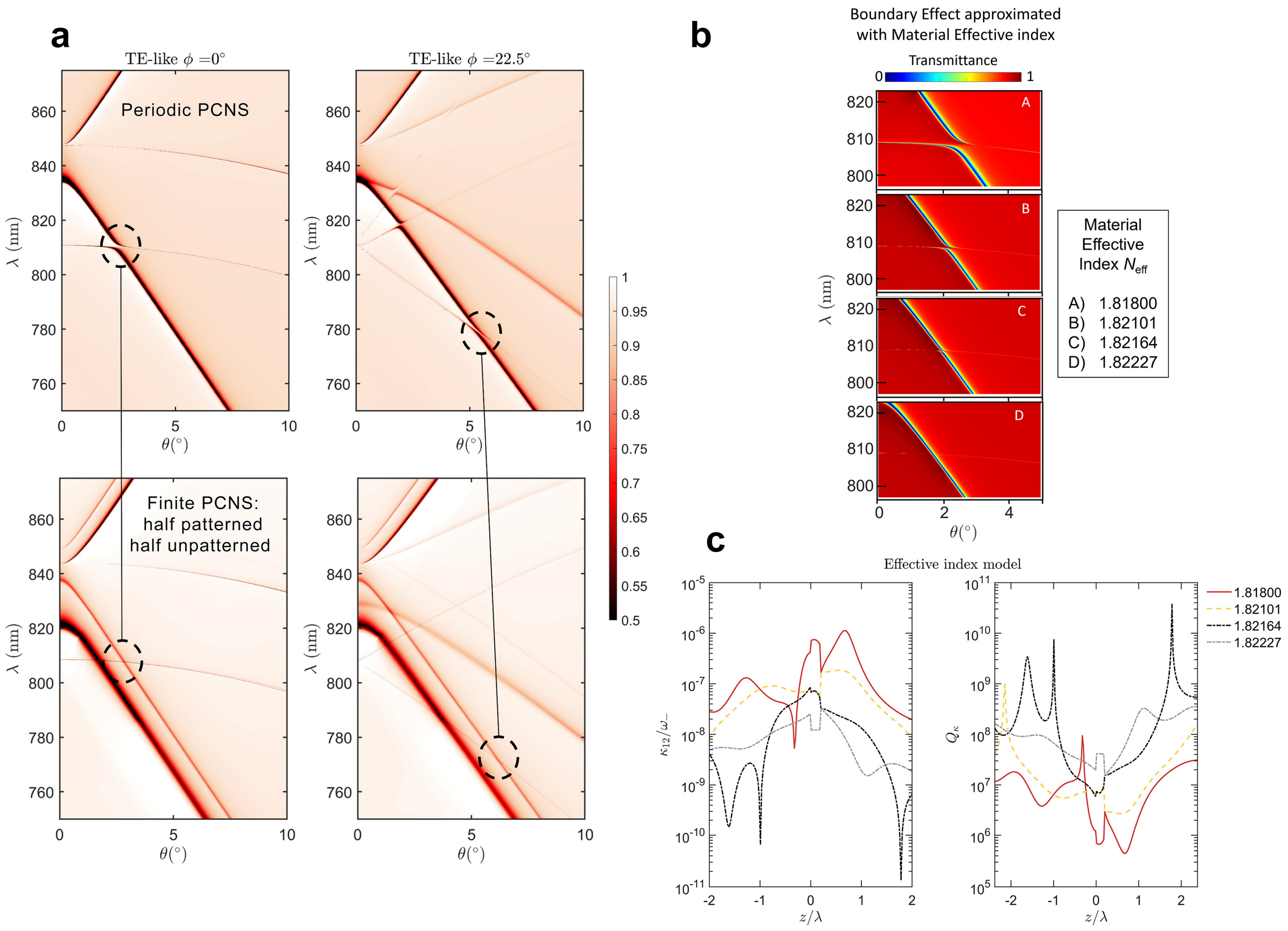

Supplementary Fig. 2 | a, RCWA simulations for periodic PCNS (top) and FDTD finite PCNS with uniform slab (bottom). The spectral separation between modes 1 and 2, which measures their near-field coupling κ_{12} reduces at the boundary between the PCNS and the unform slab. **b,** RCWA TE numerical band diagrams of the PCNS as a function of the effective index N_{eff} of the PCNS material, simulating the perturbation induced at the boundary: the effect of the boundary can be approximated with an effective material index variation with RCWA in better agreement with experiment (Fig. 3a). **c,** Near-field coupling constant (real part) normalized to $\omega_- = 2\pi c/\lambda_{mode\text{-}1}$ calculated for a fixed $\theta = 3.24°$ (highest Q_r) and associated quality-factor Q_κ (as defined above) as a function of z (normalized to λ_{mode1} = 810 nm) along the z-axis and parameterized with the effective refractive index: as the bands converge with increasing effective index, the coupling constant decreases.

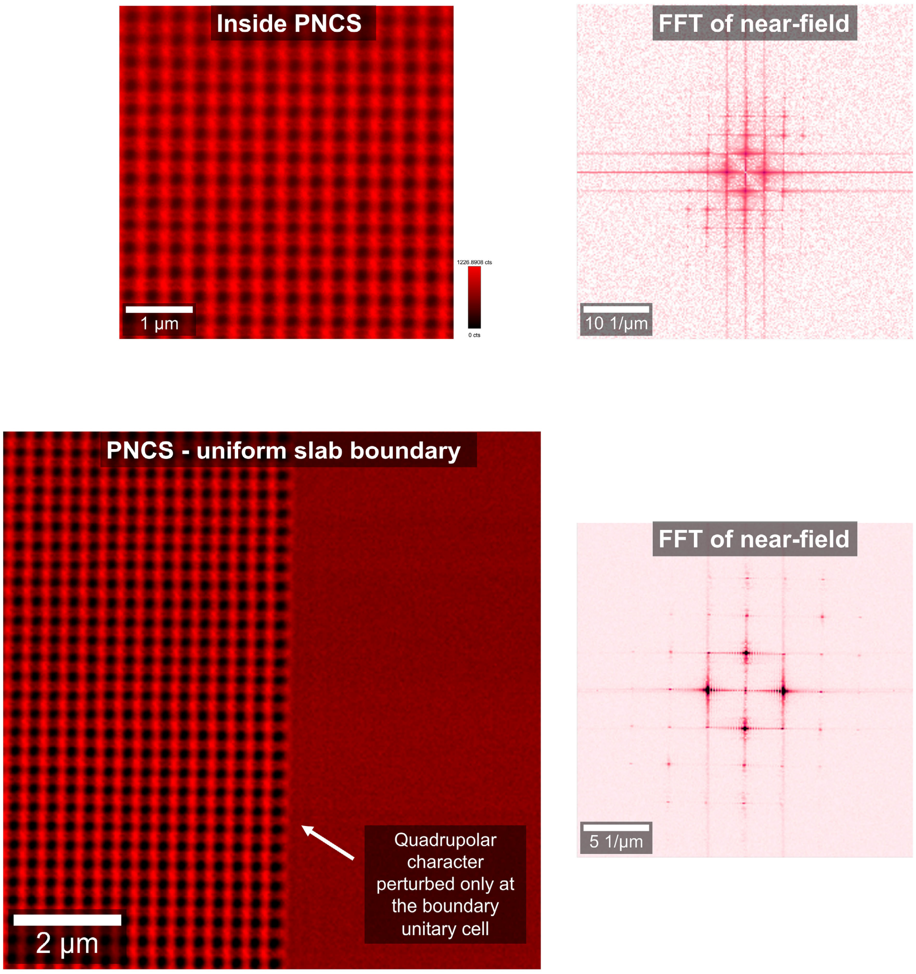

Supplementary Fig. 3 | SNOM maps of the near field excited at frequency close to the BIC, inside the PCNS (top) and close to the boundary of the finite structure (bottom), with corresponding FFT maps. The resonance is perturbed only at the last unitary cell with an intensity reduced by about 50%.

1.2 Appendix: Friedrich-Wintgen quantum model

Here, we utilize the quantum theory of Friedrich-Wintgen to demonstrate that the open-channel wave acts as a drive field in the coupled BIC equation. We directly follow the model of interfering resonances developed by Friedrich and Wintgen [1]. It is possible to write the system of three coupled equations for the open-channel wave Ψ_0 and the two closed-channel waves $\Psi_{1,2}(r)$. Let us also impose that the

waves $\Psi_{1,2}(r)$ are dominated by bound-state wave functions, *i.e.* $\Psi_1 = A\phi_1$ and $\Psi_2 = B\phi_2$, where $\phi_{1,2}$ are solutions of the uncoupled closed-channel Hamiltonians:

$$\left(-\frac{\hbar^2}{2M}\frac{d^2}{dr^2} + V_{11}\right)\phi_1 = E_1\phi_1, \tag{a1}$$

$$\left(-\frac{\hbar^2}{2M}\frac{d^2}{dr^2} + V_{22}\right)\phi_2 = E_2\phi_2. \tag{a2}$$

For $i, j \in \{0,1,2\}$, the coupled wave equations are:

$$\left(-\frac{\hbar^2}{2M}\frac{d^2}{dr^2} + V_{ii}\right)\Psi_i + \sum_{j\neq i} V_{ij}\Psi_j = \mathcal{E}\Psi_i. \tag{a3}$$

Considering that the uncoupled waves satisfy the Hamiltonians (a1) and (a2), we can write the Eq.(a3) explicitly for each Ψ_i as

$$\left(-\frac{\hbar^2}{2M}\frac{d^2}{dr^2} + V_{00}\right)\Psi_0 + AV_{01}\phi_1 + BV_{02}\phi_2 = \mathcal{E}\Psi_0 \tag{a4}$$

$$AE_1\phi_1 + BV_{12}\phi_2 + V_{01}\Psi_0 = \mathcal{E}A\phi_1 \tag{a5}$$

$$BE_2\phi_2 + AV_{12}\phi_1 + V_{02}\Psi_0 = \mathcal{E}B\phi_2. \tag{a6}$$

We can now substitute Eq. (31) in (30), which becomes

$$\left(E_1 + \frac{V_{12}^2}{\mathcal{E}-E_2}\right)A\phi_1 + \left(V_{01} + \frac{V_{12}}{\mathcal{E}-E_2}V_{02}\right)\Psi_0 = \mathcal{E}A\phi_1, \tag{a7}$$

in which $\mathcal{E}$ is the solution for the coupled wave $\Psi_1 = A\phi_1$ and two coupling terms with the open channel Ψ_0 appear: the first is the direct coupling V_{01} of the closed-channel wave Ψ_1 to the open-channel wave Ψ_0, whereas the second is provided by the direct coupling V_{02} of the closed-channel wave Ψ_2 to Ψ_0, where the balance prefactor $V_{12}/(\mathcal{E} - E_2)$ is given by the ratio between the closed-channel coupling V_{12} and the distance between the final energy $\mathcal{E}$ and the uncoupled-wave energy E_2. To estimate these terms, let us calculate the evolution of the resonances and their linewidth according to Friedrich-Wintgen's theory.

Eq. (a3) for Ψ_0 can be integrated considering the Green's function G

$$G \equiv \left(\mathcal{E} + \frac{\hbar^2}{2M}\frac{d^2}{dr^2} - V_{00}\right)^{-1}, \tag{a8}$$

from which

$$\Psi_0(r) = \phi_0(r) + A\int_0^\infty G(r,r')V_{01}(r')\phi_1(r')dr' + B\int_0^\infty G(r,r')V_{02}(r')\phi_2(r')dr', \tag{a9}$$

where ϕ_0 is the uncoupled wave solution of the open channel. Following the calculation in [1], it is possible to solve the system of Eqs. (a5) and (a6) for the coefficients A and B that define the perturbation of the uncoupled closed-channel wave functions $\phi_{1,2}$ when coupled to the open channel. It is useful to write all the coupling terms explicitly as

$$W_{10} = \phi_1 V_{01} \phi_0$$
$$W_{20} = \phi_2 V_{02} \phi_0$$
$$M_{12} = \phi_1 V_{12} \phi_2$$
$$G_{11} = \phi_1 V_{01} G V_{01} \phi_1 \quad \text{(a10)}$$
$$G_{22} = \phi_2 V_{02} G V_{02} \phi_2$$
$$G_{12} = \phi_1 V_{01} G V_{02} \phi_2$$
$$W_{12} = M_{12} + G_{12}.$$

It follows that

$$A = \frac{T_2 W_{10} + W_{12} W_{20}}{T_1 T_2 - W_{12}^2}$$
$$B = \frac{T_1 W_{20} + W_{12} W_{10}}{T_1 T_2 - W_{12}^2}, \quad \text{(a11)}$$

in which $T_i = \mathcal{E} - \epsilon_i$ and $\epsilon_i = E_i + G_{ii}$, $i \in \{1,2\}$. The energy solutions of the closed-channel coupled waves are

$$\mathcal{E}_{1,2} = \frac{\epsilon_1 + \epsilon_2}{2} \pm \sqrt{\frac{(\epsilon_1 - \epsilon_2)^2}{4} + W_{12}^2}, \quad \text{(a12)}$$

and thus obey the strong coupling splitting $2|W_{12}|$, given by the interaction energy. If the waves were photons, Eq. (a12) would provide the frequency splitting in the dispersion bands of the interfering modes. This means that the splitting $2|W_{12}|$ is provided by the intercoupling M_{12} (near-field coupling) plus an additional energy shift mediated by the coupling to the open channel G_{12} (far-field coupling). The linewidths of the coupled waves are given by the following relation:

$$\Gamma_{1,2} = \frac{1}{\mathcal{E}_{1,2}} \left[W_{10}^2 + W_{20}^2 \pm \frac{(W_{10}^2 - W_{20}^2)(\epsilon_1 - \epsilon_2)/2 + 2 W_{10} W_{20} W_{12}}{\sqrt{(\epsilon_1 - \epsilon_2)^2/4 + W_{12}^2}} \right], \quad \text{(a13)}$$

which can be simplified by rescaling the energy reference such that $(\epsilon_1 + \epsilon_2)/2 = 0$ for a more clear-cut depiction of the dependence as a function of $\Delta\epsilon = \epsilon_1 - \epsilon_2$. The plot in **Supplementary Fig. 4** shows the evolution of the resonances and their linewidths as a function of $\Delta\epsilon$ for a set of coupling parameters. It is shown that the internal coupling energy $W_{12} = M_{12} + G_{12}$ must be larger than the external couplings to the open channel W_{10} and W_{20} to have nonoverlapping resonances. This means that $\mathrm{M}_{12} = \phi_1 V_{12} \phi_2$ must be larger than $G_{12} = \phi_1 V_{01} G V_{02} \phi_2$ in order to satisfy this condition. In addition, the broadening of the energy linewidth Γ_2 strongly depends on the relative strength between internal and external couplings. Only if W_{12} is much larger than the external coupling terms, Γ_2 does not show a large broadening. In this case, it is possible to write, nearby the avoided crossing point, that the

expectation value of $\phi_2 V_{12} \phi_1 = W_{12} - G_{12} \simeq W_{12}$ and $\mathcal{E}_1 - E_2 \simeq W_{12}$ (from Eq. a12) when the splitting is of the order of the linewidth of the uncoupled waves and the resonances do not overlap. Let us consider the coupling strengths V_{01} and V_{02} multiplying Ψ_0 in Eq. (a7). The terms in Eq. (a7) have the following correspondence: $V_{12} \to \kappa_{12}$, $\Psi_0 \to s_+$, $V_{01} \to \sqrt{2/\tau_{r1}}$ and $V_{02} \to \sqrt{2/\tau_{r2}}$. The second coupling term to the open channel in Eq. (a7) is of the order of V_{02} since the prefactor $V_{12}/(\mathcal{E}_1 - E_2)$ provides an expectation value of the order of unity. Thus, Eq. (a7) shows two driving fields for wave ϕ_1 of comparable strength, which are related to the coupling to the open channel Φ_0 not only by ϕ_1 *via* V_{10}, but also by ϕ_2 *via* V_{20}. This agrees with the dark mode field expression found in the main paper by TCMT, Eq. (35), which includes the drive mediated by the bright partner.

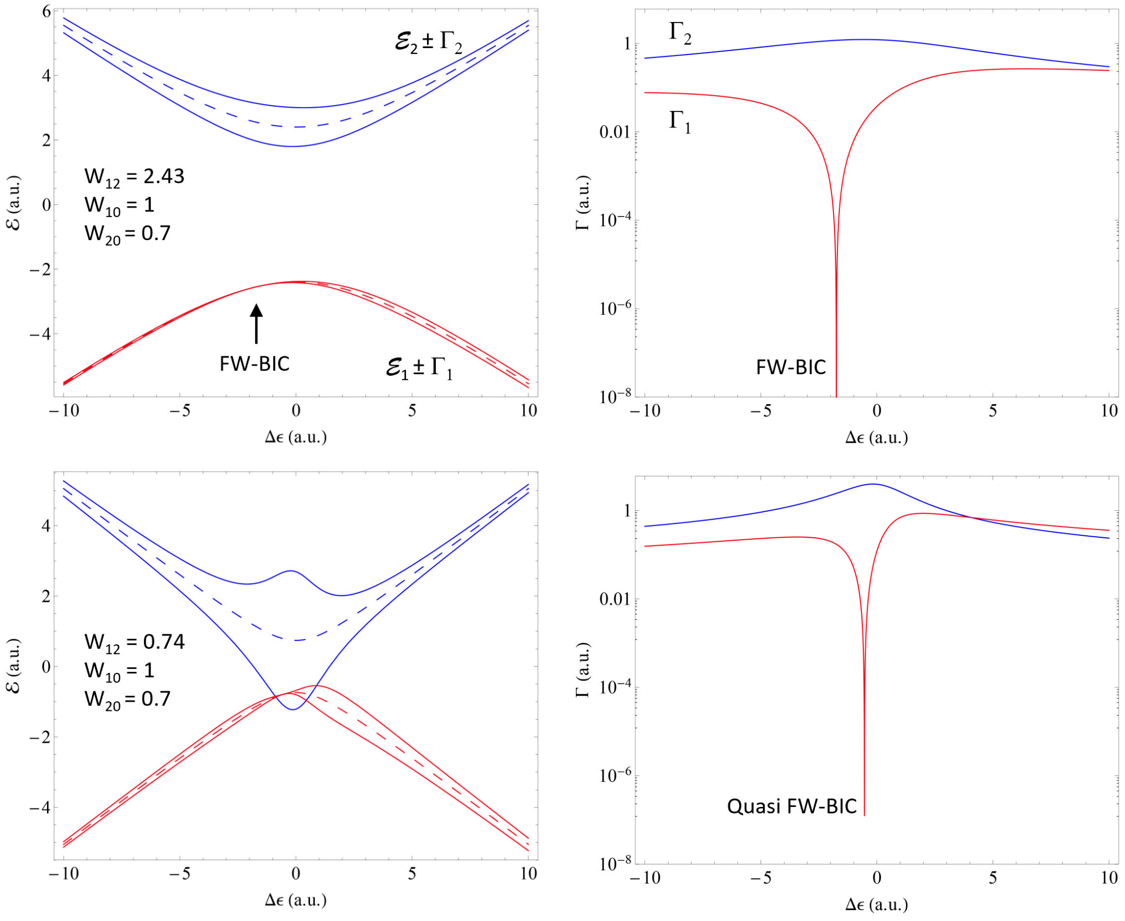

Supplementary Fig. 4 | Theoretically calculated coupled resonance evolution for the coupling coefficient indicated in the graphs: when W_{12} is of the order of W_{10} or W_{20}, the resonance broadening gives a large overlap at the avoided crossing point $\Delta\epsilon = 0$. Right panels show in detail the linewidths for the same parameters as in the left panels.

1.3 Quasi symmetry-protected BICs *versus* Friedrich-Wintgen quasi-BICs

Quasi symmetry-protected (SP)-BICs, *i.e.* modes with high but finite Q-factor induced by symmetry protection, are not produced by coupled mode interference as in the FW two-wave scheme and as such are isolated resonances in the dispersion diagram obeying the conventional coupling mechanism discussed in Methods, Sec. 1.1. Therefore, a good far-field coupling can be achieved with these dark modes after breaking the symmetry of the structure [2].

2 Synthesis of Upconversion photoluminescence (UCPL) NPs

2.1 Materials

1-Octadecene (ODE, 90 %), oleic acid (OA, 90 %), yttrium acetate hydrate ($Y(CH_3CO_2)_3 \cdot xH_2O$; 99.9%), erbium acetate hydrate ($Er(CH_3CO_2)_3 \cdot xH_2O$; 99.9 %), thulium acetate hydrate ($Tm(CH_3CO_2)_3 \cdot xH_2O$; 99.9%), gadolinium acetate hydrate ($Gd(CH_3CO_2)_3 \cdot xH_2O$; 99.9 %), europium acetate hydrate ($Eu(CH_3CO_2)_3 \cdot xH_2O$; 99.9%), yterbium acetate hydrate ($Yb(CH_3CO_2)_3 \cdot xH_2O$; 99.9%), neodymium acetate hydrate ($Nd(CH_3CO_2)_3 \cdot xH_2O$; 99.9%), sodium hydroxide (NaOH; >98%), ammonium fluoride (NH_4F;>98%), cyclohexane (>98%), ethanol (>99%), and methanol (>99%) were all purchased from Sigma–Aldrich and used as received without further treatment.

2.2 Synthesis of $NaErF_4$ core nanocrystals

In a typical experiment, an aqueous solution (2 mL) of $Er(CH_3CO_2)_3$ with a total lanthanide content of 0.4 mmol was added to a 50 mL flask charged with 7mL ODE and 3 mL OA. After heating at 150 °C for one hour, the water was removed. Then the mixture was cooled to 50 °C, and a methanol solution containing 1.6 mmol NH_4F and 1 mmol NaOH was added. After stirring for 30 min, the solution was heated and pumped at 100 °C for 15 min to remove the methanol. Then, the mixture was heated to 290 °C and maintained at this temperature for 2.5 h under argon protection. After cooling, the products were collected by centrifugation with ethanol. Finally, the precipitates were redispersed in 1 mL of cyclohexane for further shell growth.

2.3 Synthesis of $NaErF_4$@$NaYF_4$ core-shell nanocrystals

A 2 mL aqueous solution of $Y(CH_3CO_2)_3$ was added to a 50 mL flask. Then 7 mL of ODE and 3 mL of OA were added to the solution. The solution was then heated at 150 ºC for 1 h to remove the water and form the lanthanide complex. After cooling to 80 ºC, the $NaErF_4$ core nanocrystals dispersed in cyclohexane were added to the obtained solution. The cyclohexane was then removed by keeping the mixture at the same temperature for 30 min. Then the mixture was cooled to 50 ºC and 1.6 mmol NH4F and 1 mmol NaOH solution in methanol solution were added. After stirring for 30 min, the mixture was heated to 290 ºC and kept under argon gas flow for three hours. After cooling down to room temperature, the core-shell nanocrystals were collected by centrifugation with ethanol. Finally, the as-obtained core-shell structured nanocrystals were washed with ethanol and then redispersed in 4 mL cyclohexane.

2.4 Synthesis of $NaGdF_4$:Nd/Yb(40/5%) @$NaGdF_4$:Yb/Tm(49/1%) @$NaGdF_4$:Eu(15%) core-shell-shell nanocrystals

The experimental details for the synthesis of $NaGdF_4$:Nd/Yb(40/5%)@$NaGdF_4$:Yb/Tm(49%/1%)@$NaGdF_4$:Eu(15%) core-shell-shell nanocrystals are very similar to that for $NaErF_4$@$NaYF_4$ core-shell nanocrystals. $Nd(CH_3CO_2)_3 \cdot xH_2O$, $Yb(CH_3CO_2)_3 \cdot xH_2O$, and $Gd(CH_3CO_2)_3 \cdot xH_2O$ (molar ratio of Nd/Yb/Gd is 40/5/55) are used as the lanthanide ion sources for synthesizing $NaGdF_4$:Nd/Yb(40/5%) core nanocrystals. Then, $Tm(CH_3CO_2)_3{\cdot}xH_2O$, $Gd(CH_3CO_2)_3{\cdot}xH_2O$, $Yb(CH_3CO_2)_3{\cdot}xH_2O$ (Yb/Tm/Gd=49/1/50) are used for the preparation of the shell precursor in OA/ODE solution.

By adding the $NaGdF_4$:Nd/Yb(40%/5%) core nanocrystals in the shell precursor, $NaGdF_4$:Nd/Yb(40/5%) @$NaGdF_4$:Yb/Tm (49/1%) core-shell nanostructures can be prepared following the experimental processes described above. The coating of the outermost shell of $NaGdF_4$:Eu can be achieved with a mixed OA/ODE solution containing $Gd(CH_3CO_2)_3{\cdot}xH_2O$ and $Eu(CH_3CO_2)_3{\cdot}$xH2O (molar ratio of Eu/Gd is 15/85) as the shell precursor and previously prepared $NaGdF_4$:Nd/Yb(40/5%)@$NaGdF_4$:Yb/Tm (49/1%) nanocrystals as the core product.

3 Characterization of the as-produced NPs

A JEOL-1400 TEM, operating at an acceleration voltage of 100 kV, was used to characterize the morphology of prepared core-shell upconversion nanocrystals (**Supplementary Fig. 5**). An acceleration voltage of 200 kV was used for high-resolution TEM imaging with the same instrument. UV/Vis absorption spectra of the Er-activated core-shell upconversion nanocrystals were acquired using a SHIMADZU UV-3600 UV/Vis-NIR spectrophotometer with a cyclohexane solution as the reference. Upconversion luminescence spectra were recorded using an Edinburgh FSP-920 spectrometer in conjunction with a continuous-wave 808 nm laser (MDL-III-808 nm). The power-dependent upconversion luminescence data were collected by varying the excitation power of the 808-nm laser while all other parameters were fixed (**Supplementary Fig. 5**).

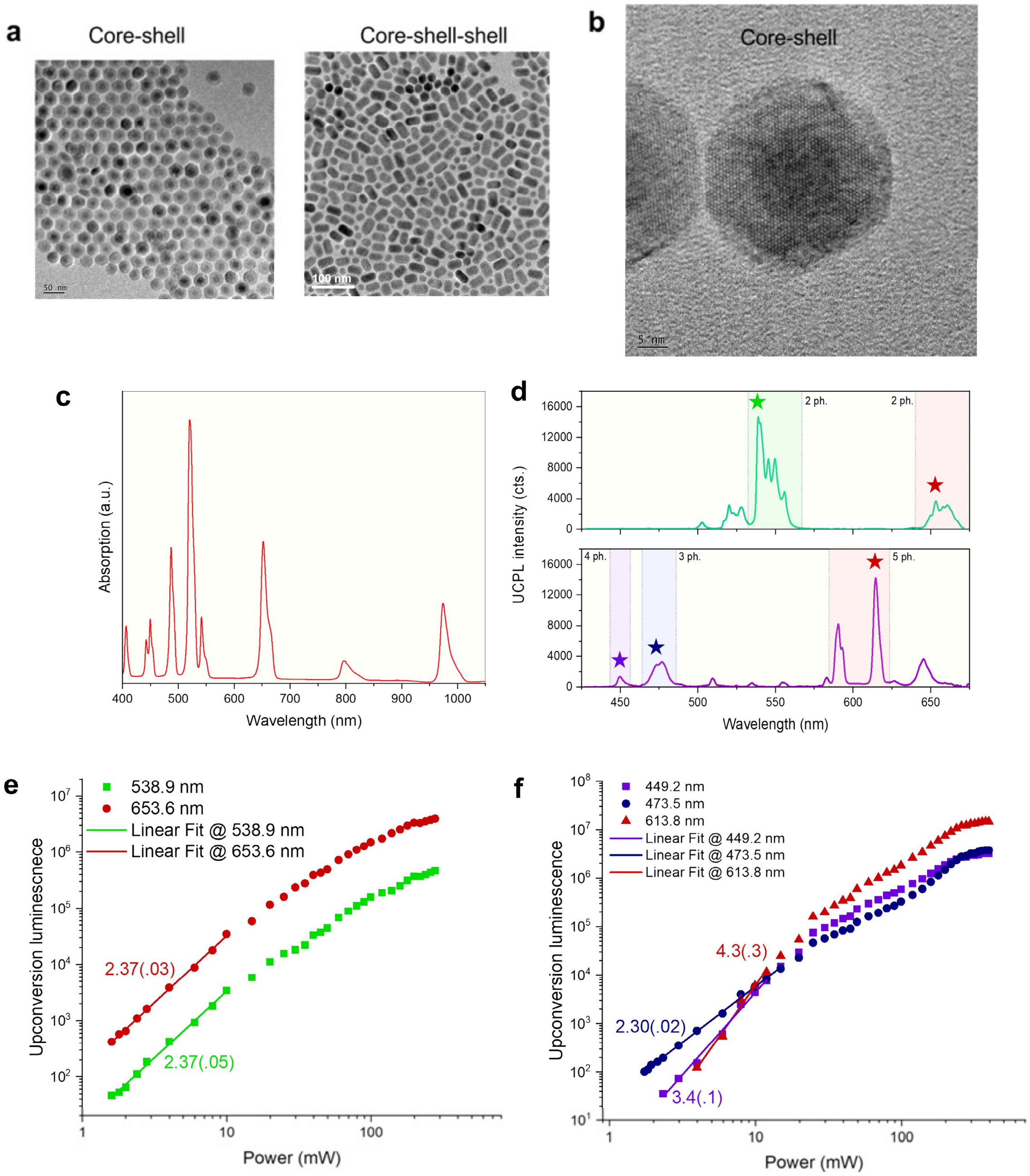

Supplementary Fig. 5 | a and **b,** TEM characterizations of core-shell and core-shell-shell NPs, respectively. **c,** Optical absorption spectrum of core-shell NPs. **d,** Detailed spectral features of UCL from both core-shell and core-shell-shell NPs. **e,** and **f,** Incident power dependence (at λ_{exc} = 808 nm) of the emission lines marked in **d**.

4 Experimental samples

4.1 PCNS fabrication

The Si_3N_4 film was deposited on a quartz substrate by plasma-enhanced chemical vapor deposition. The film was patterned via electron beam lithography and a coupled plasma etching process. The whole procedure followed an established protocol [3]. The slab structural parameters (hole diameter, lattice period, film thickness, and refractive index dispersion) were set to match the optimal resonance conditions iteratively in accordance with the formulated numerical predictions as well as the experimental refractive index dispersion.

4.2 Upconversion Nanoparticles (UCNPs) deposition and morphology

The upconversion NPs film was deposited onto the PCNS by spin-coating 100 mL of NP suspension at 1500 rpm, C[core-shell] = 10 mg/mL, C[core-shell-shell] = 5 mg/mL), with an acceleration ramp of 1 s and a duration of 60 s. In **Supplementary Fig. 6**, the conformal layer of UCNPs coating the PCNS was investigated by atomic force microscopy (XE-100 AFM, Park Systems) operating in non-contact mode using a reflectively coated cantilever (Park, resonance frequency 300 kHz) at a scan frequency of 0.5 Hz. AFM images were analyzed using XEI 1.8.1.build214 software (Park Systems) to evaluate the thickness and uniformity of the NP coating (**Supplementary Fig. 6e**) to estimate the effective volume fraction interacting with the localized PCNS mode, as shown in **Extended Data Fig. 2c-d**.

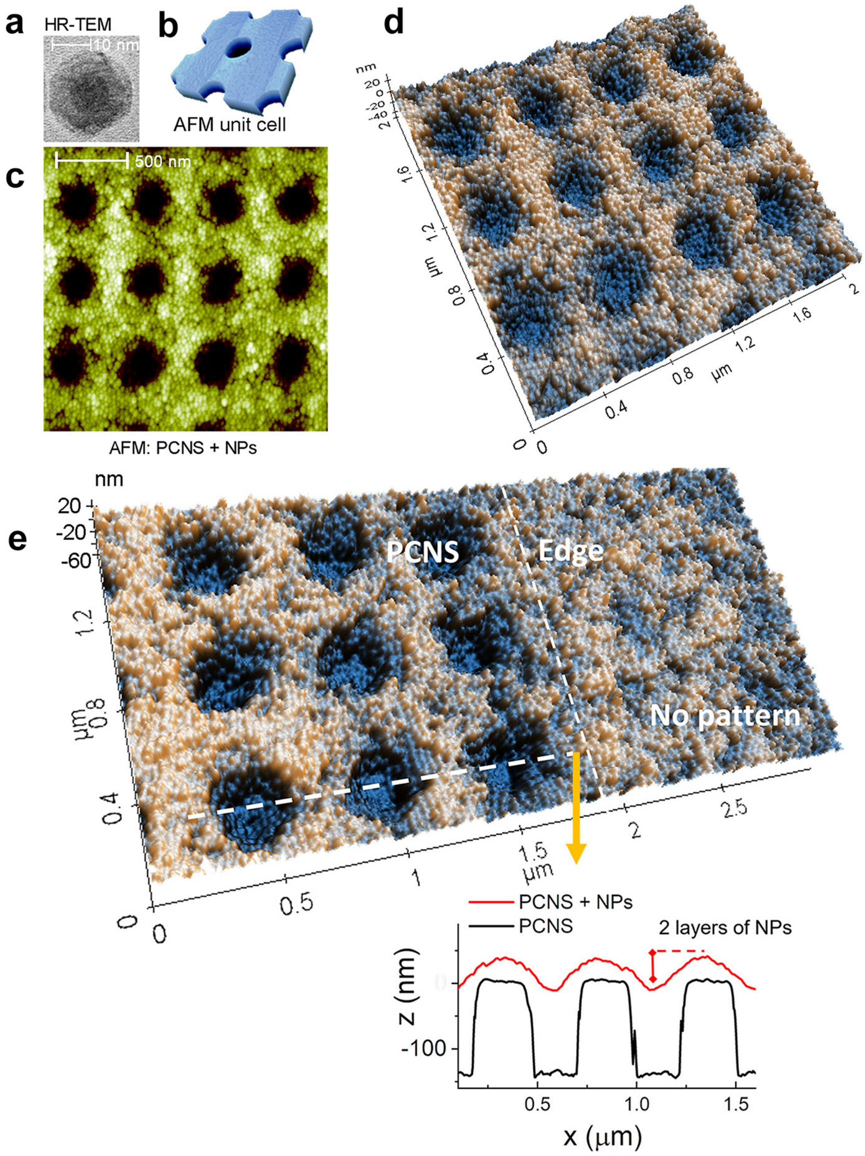

Supplementary Fig. 6| a, HR-TEM of a core-shell UCNP. **b,** Detailed topography of the PCNS unit cell. **c,** Topography of the PCNS coated with UCNPs, with an enhanced color bar in **d**. **e,** UCNP superstructure near the boundary of the patterned area with topographic profile before and after UCNP deposition.

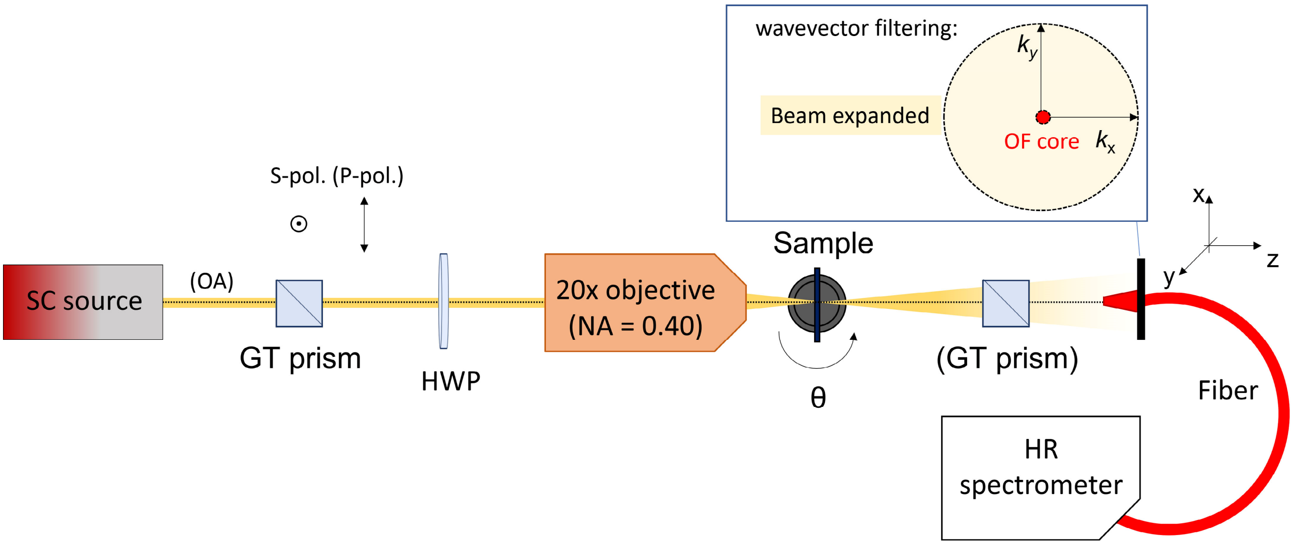

Supplementary Fig. 7 | Detailed optical setup implemented for angle-resolved transmission spectroscopy measurements. SC: supercontinuum; OA: optical axis; GT: Glan-Thomson; HWP: half-wave plate.

5 Dispersion band diagram measurements

The optical response of the PCNS before and after NP deposition was characterized by angle-resolved transmission spectroscopy using a supercontinuum laser with a beam diameter of 2 mm (NKT Photonics, SuperK EXTREME, 400-2400 nm single-mode output, 4 Watts), which was appropriately polarized using a Glan-Thompson (GT) prism and a half-wave plate mounted in cascade. The beam was focused onto the sample using a 20x Mitutoyo Plan Apo NIR objective (NA = 0.40) with long-working distance (20 mm) and infinity correction, with the same spot-size used for optical pumping of upconversion. Wavevector dispersion was acquired by rotating the sample *via* an externally-driven system with an angular step of 0.05°. The whole procedure was managed *via* a custom-built MathWorks MATLAB routine for acquisition and band reconstruction. Where appropriate, another GT prism was employed for output prior to signal collection. A high-resolution spectrometer was used for spectral detection (Ocean Optics HR4000). An additional filtering process *via* fast Fourier transform in MATLAB was used to subtract the background signal and remove interference fringes. The schematic of the optical setup is reported in **Supplementary Fig. 7**. Far from the sample, the input beam expansion allows spatial filtering of the wavevector components in the core of the optical fiber along the propagation axis. The angular

range of wavevectors filtered in this way for detection is given by the ratio of the core diameter and the expanded beam diameter, and is approximately $5.72° \times \frac{0.2\ \mathrm{mm}}{50\ \mathrm{mm}} = 0.023°$, where the core size is 200 mm and the beam size is expanded up to 5 cm. This approach is equivalent to using a collimated source with a divergence of less than 0.4 mrad with the advantage of micrometric spatial resolution of ~6 μm for point-wise scanning of the sample response near the edge, with the same spot-size used for optical pumping of upconversion.

For accurate linewidth measurements, the local spectrum was reconstructed by scanning the 4-nm-wide laser input wavelength at a step of 0.1 nm/s through a tunable acoustic-optic filter (Select, NKT Photonics). The reflectance resonance appears as a dip in the transmission envelope with the input peak profile. The resonance is hence deconvolved from the transmittance with a peak-find regression, providing the accurate linewidth fit with a resolution < 0.1 nm. Alternatively, an optical spectrum analyzer, Ando AQ6317B (600-1750 nm), with a resolution of 0.01 nm was used for specific measurements when needed.

Supplementary Fig. 8 shows angle-resolved TM transmission bands of the sample, indicating experimental SP- and FW-BIC positions.

Supplementary Fig. 9 shows that FW-BIC is present for both input TE (vertical polarization) and TM (horizontal polarization) used to excite the response transmittance. The finite slab geometry actually induces a modal vector field with strong cross-coupling between the purely transverse electric and purely transverse magnetic character in the TE-like band. The FW-BIC is located at the crossing point with the largest cross-coupling and can be excited with both input polarizations.

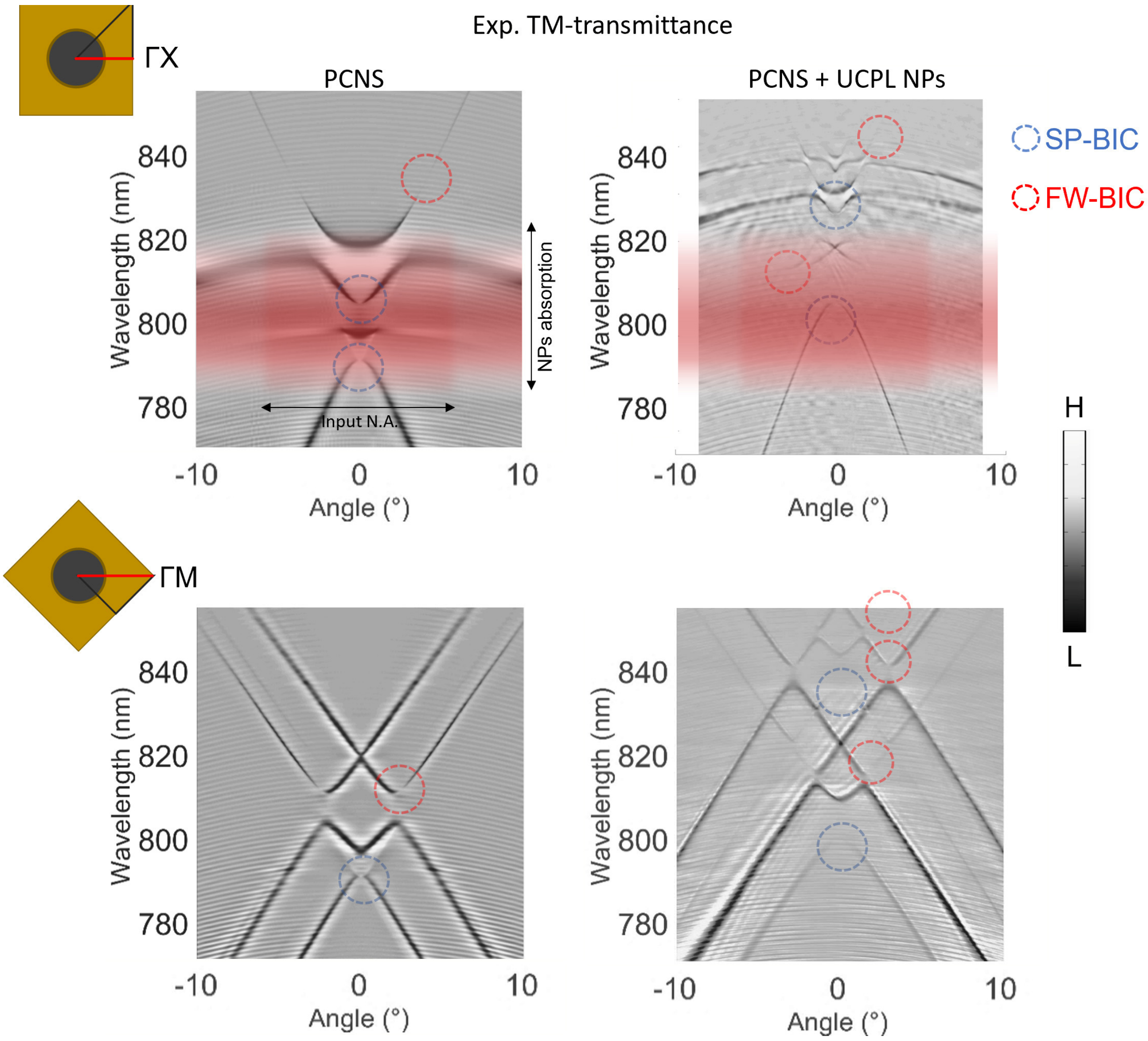

Supplementary Fig. 8 | Experimental TM dispersion bands along the high-symmetry directions ΓX and ΓM of PCNS and PCNS + UCNP samples. Herein, the traceable spectral intervals, dictated by the NP absorption range, as well as the input N.A. of the focusing objective, are also highlighted.

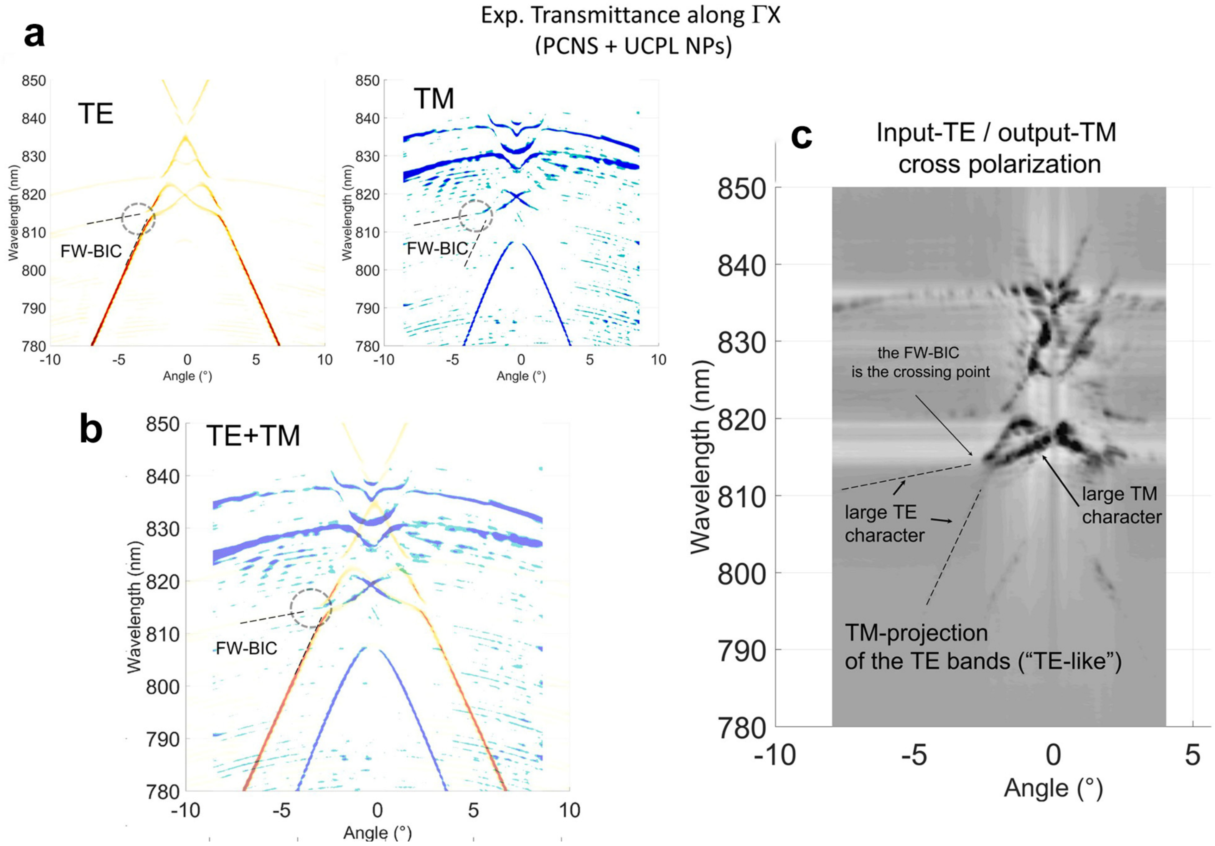

Supplementary Fig. 9 | Experimental dispersion bands along Γ*X* of PCNS + UCNP samples. a, TE and TM dispersion diagrams with white-to-red and white-to-blue colorbars, respectively, show that FW-BIC is present for both of the input field polarization used to excite the transmittance. **b,** Overlap of TE and TM dispersion diagrams. **c,** Cross-polarized band diagram showing that the TE-like band has a strong TM character given the PCNS thin slab geometry. The FW-BIC is located at the crossing-point between TE and TM prevalent characters. In other words, the FW-BIC mode is excited with both input polarizations.

6 UCPL measurements: Experimental interrogation and detection

Upconversion photoluminescence studies were carried in transmission. The implemented setup is depicted in detail in **Supplementary Fig. 10**. The pump beam (Chameleon Ultra II Ti:Sa laser, pulse of 140 fs, repetition rate 80 MHz) was focused onto the sample close to normal incidence using a long-working distance 20x Mitutoyo Plan Apo NIR infinity-corrected objective (NA = 0.40) (beam waist $w_o = 3$ μm, beam spot: 6 μm). The upconverted signal was collected with a 50x Mitutoyo Plan Apo NIR infinity corrected objective (NA = 0.42), guided to an optical fiber and dispersed in a high-resolution spectrometer (Ocean Optics HR4000). Proper optical filter components were utilized to reduce pumping residue. In-plane BIC-driven supercollimated radiation was collected by setting the optical fiber orthogonal to the incident direction in proximity to the edge of the quartz slide. A Thorlabs DCC1240M camera was used to optimize the pump focus on the sample and to image the output beam.

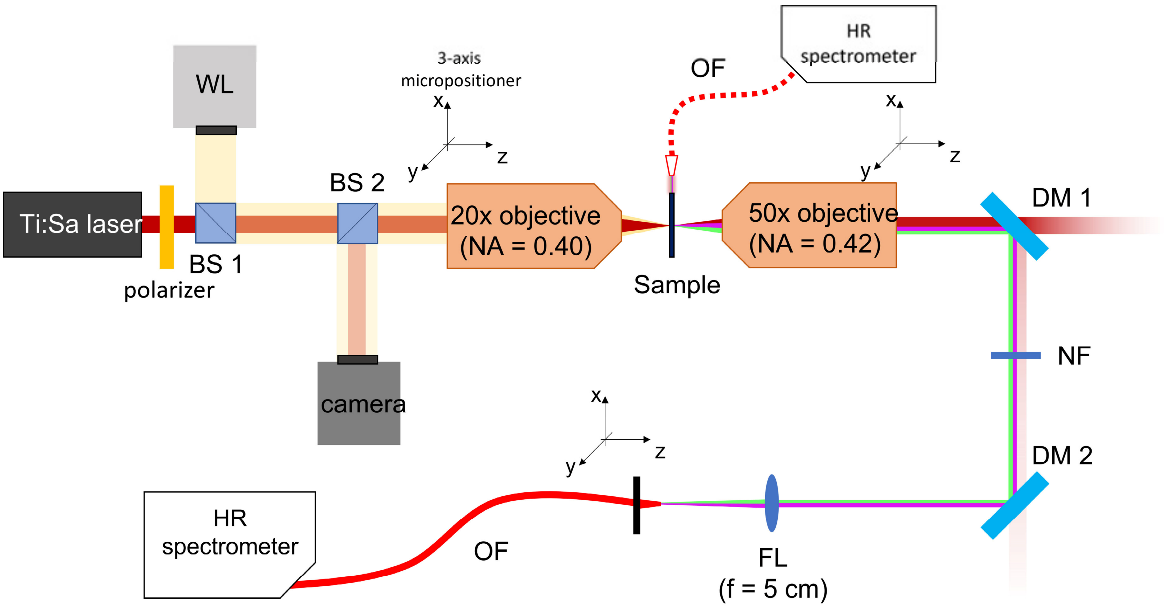

Supplementary Fig. 10 | Detailed optical setup for BIC-enhanced UCPL experiments. SC: supercontinuum; OA: optical axis; GT: Glan-Thomson; NA: numerical aperture; BS: beam splitter; WL: white light; DM: dichroic mirror; NF: notch filter (center wavelength 808 nm); FL: focusing lens.

Excitation quality factor. The pump had a pulse duration $T = 140$ fs. Therefore, an excitation quality

factor at 810 nm of Q_{ex} = 170 can be readily determined using the hyperbolic secant approximation for the bandwidth [2]. Q_{ex} should be approximately three times the quality factor of the resonance to transfer the input energy to the system more efficiently. However, this is not a trivial task for a resonator with high Q and an unchirped source. Therefore, a certain amount of uncoupled light is expected in this system, but that can be treated in other schemes. Since the relevant resonance in the case of edge emission is the bright mode with an average Q_{R2} = 213, the optimal Q_{ex} should be approximately 600. The estimated value of 170 is only about one third of the optimum, but not far from the best value. However, it is worth mentioning that the conclusion regarding the enhancement factor is not affected by this circumstance since it is compensated by comparing the signal between the structured region and the unstructured film.

7 Enhancement factor estimation

Supplementary Fig. 11a shows the scheme of collection for forward scattering measurements of UCPL radiation. **Supplementary Fig. 11b** schematically shows the different emission and collection properties associated with the edge-mode excitation. As can be seen in **Supplementary Fig. 11a**, the detection scheme for UCPL measurements of the PCNS coated with NPs and the bulk reference of NPs is exactly the same, except for the number of NPs involved. The number of UCNPs in the scattering area $A_i = \pi w_o^2$, defined by the focused laser beam waist $w_o = 3$ μm, can be easily estimated by considering the geometry of the NP distribution in the unit cell and then summing over the number of unit cells in the scattering area. The NP diameter is 20 nm on average and occupies a nearly spherical volume $V_{NP} \simeq \frac{4}{3}\pi 10^3$ nm^3. Two layers of thickness δh cover an area given by $a^2 = 551^2$ nm^2 giving the volume $a^2\delta h$ plus the further contribution of the filled hole or radius $r = 130$ nm, giving the volume $V_h = \pi r^2(h - \delta h)$, with $h = 130$ nm, thus contributing to the whole occupied volume in the unit cell $V_u = a^2\delta h + V_h$, reduced by random close packing fraction $\rho = 0.64$. The number of NPs excited on the PCNS is thus

$$N_a = \frac{A_i}{a^2}\frac{\rho V_u}{V_{NP}} = \frac{28\ \mu m^2}{0.3\ \mu m^2} \times \frac{0.64\times(12\times10^{-3}+5.8\times10^{-3})\ \mu m^3}{0.0042\times10^{-3}\ \mu m^3} = 2.53 \times 10^5. \quad (7.1)$$

The number of NPs in the bulk, N_b, is instead given by the volume of the Gaussian beam in the $x - y$ plane integrated in the range $(-z/2, +z/2)$ over an axial length z corresponding to the deposited NP film thickness of 15 μm. The Rayleigh length of the beam is $z_R = 15\mu$m. Thus, the required volume is $V_b = 624$ μm^3. Thus, $N_b = \rho\frac{V_b}{V_{NP}} = 9.5 \times 10^7$. The ratio N_b/N_a is thus 375 and represents the

normalization factor to account for the different number of NPs involved in the measurements.

Forward scattering. The UCPL intensity measured on the PCNS in forward scattering, as depicted in **Supplementary Fig. 11a**, is $I_a = 3.2 \times 10^3$ counts, with an input power P_a = 100 mW and $\Delta t_a = 10$ s. On the bulk reference, measured in forward scattering, the intensity is $I_b = 0.8 \times 10^3$ counts, with an input power P_b = 100 mW and $\Delta t_b = 2$ s. This gives:

$$EF_{f,exp} = \frac{I_a}{I_b}\frac{\Delta t_b}{\Delta t_a}\frac{P_b}{P_a}\frac{N_b}{N_a} = 300. \quad (7.2)$$

Side-beam collection. The UCPL intensity of the edge emission from the PCNS, as sketched in **Supplementary Fig. 11**, is $I'_a = 1.6 \times 10^4$ counts at 540 nm, detected with an integration time $\Delta t'_a = 0.2$ s and an input power power P_a = 10 mW. **Supplementary Fig. 12** shows the reproducibility on several samples, the statistical analysis at 540 nm for evaluating the enhancement factor, and the relative intensity of the principal upconversion emission peaks. Using the above equation (same bulk reference), the directional enhancement factor results

$$EF_{d,exp} = \frac{I'_a}{I_b}\frac{\Delta t_b}{\Delta t'_a}\frac{P_b}{P'_a}\frac{N_b}{N_a} = 0.75 \times 10^6. \quad (7.3)$$

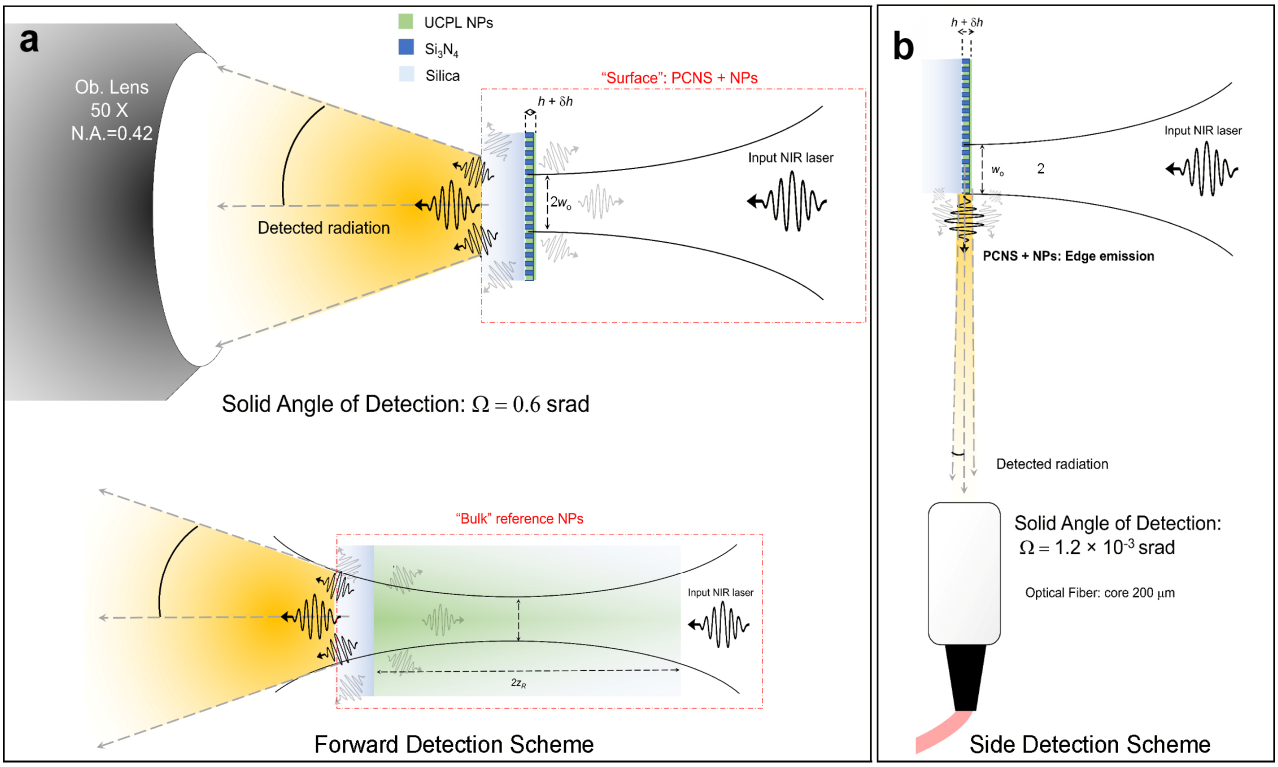

Supplementary Fig. 11 | a, Layout of UCPL detection (orange beam) with the objective lens. The detection is the same in the case of emission from the PCNS (coated with NPs, top scheme) and the bulk sample of NPs (bottom scheme): the number of NPs contributing to the signal depends only on the excitation volume, which is a surface-like excitation in the top scheme and a bulk volume in the bottom scheme. **b,** UCPL radiation is highly directional under the edge-mode excitation at the boundary of the PCNS and collimates within a tiny solid angle fully covered by an optical fiber.

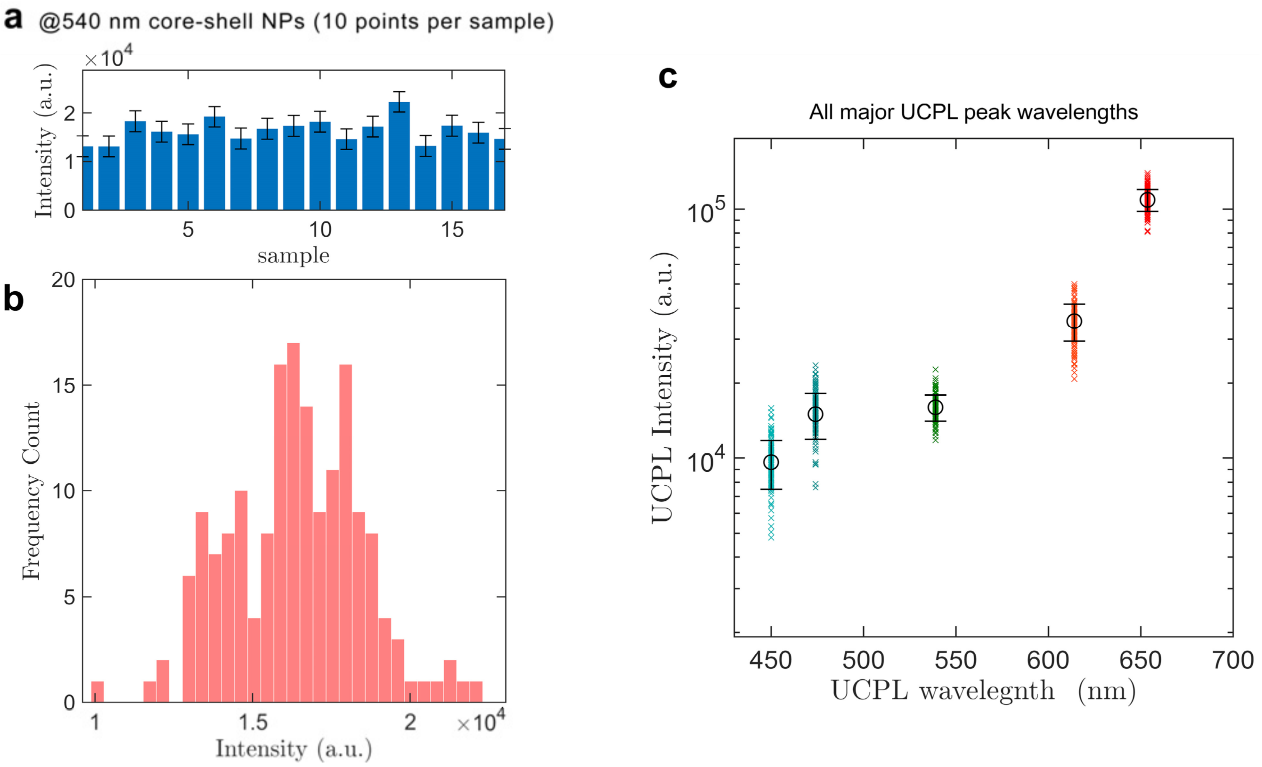

Supplementary Fig. 12 | a, UCPL emission at 540 nm as a function of the PCNS number (10 points for each sample). **b,** Histogram of the intensity distribution (UCPL peak @540 nm) from core-shell NPs used for EF calculation. c, Intensity distribution of emission achieved when scanning the input beam along the full boundary of PCNS for all major upconversion wavelengths.

Radiance correction factor. The high directivity of the side beam (scheme in **Supplementary Fig. 11b**) confines the radiation within a solid angle $\Omega_1 \simeq 1.2 \times 10^{-3}$ srad, which covers most of the emitted light collected by the optical fiber with a core of 200 μm. This means that the fraction of the emitted light collected is $\eta_1 \simeq 1$. On the contrary, the bulk emission is collected with a solid angle $\Omega_2 \simeq 0.6$ srad (**Supplementary Fig. 11a**), which implies that the fraction of the ideally isotropic bulk emission that covers the full solid angle $\Omega_{sphere} = 4\pi$ and is actually collected is $\eta_2 = 0.6/4\pi = 0.048$. The value $EF_{d,exp}$ must be corrected considering the ratio $\eta_2/\eta_1 \simeq \eta_2$, so that the actual enhancement factor is

$$EF_{exp} = \frac{\eta_2}{\eta_1} E_{d,exp} = 0.048 \times (0.75 \times 10^6) = 3.6 \times 10^4. \quad (7.4)$$

Extracted Enhancement Factor parameters from Q factors. To compare the expected enhancement factor EF_{th} with the experimental factor, we consider that the power exponent s is itself a function of the local field. In colloidal samples we measured s = 1.2 at an energy pulse of 6.25 nJ, while we found s = 1.2 at 12.5 pJ on the PCNS at 540 nm. At the edge, we measured s = 1.0 for low incident power; however, saturation caused it to change to 0.2 above 2.5 nJ pulse energy (400 kW/cm^2). Given the range of incident power used for the enhancement evaluation, we consider s =0.8÷ 1.2.

Then, we extracted the parameters used in main paper from the experimental band diagram. We recall that Q_{R1} and Q_{R2} denote the radiative quality factors of FW quasi-BIC and leaky-wave bands, respectively. The average total Q_{av} (1280) spanned by the input laser focused with the objective lens was obtained by integrating the values of Q in reciprocal space over the excitation angles. The radiative Q_{R1} of the resonator is a fast power of the incidence angle. Assuming that the intrinsic Q_I is dominated by the dissipation channel ($Q_{R1} \gg Q_a$ at the FW quasi-BIC point), the maximum of the total quality factor $Q_{max} \simeq Q_a$ is independent of the incidence angle due to material absorption and other losses. Away from the FW quasi-BIC point, the total Q_I becomes dominated by the fast decrease of Q_{R1}. The calculated Q_{R1} of the FW quasi-BIC band balances the constant Q_a that reproduces the experimental intrinsic Q_I in the dispersion curve shown in the main paper **Fig. 3d**.

The integrated radiative quality factor gives $Q_{R1,av}$ = 169*1.* The extraction efficiency is $\kappa_o \simeq 1$ at upconversion wavelengths. Using Eq. (*1*) from the main paper, based on the uncoupled resonance model and power-law scaling s in the range (0.8, 1.2), the expected average emission intensity enhancement factor EF_{th} would be

$$EF_{\text{th}} = \kappa_{\text{o}} G_{\text{av}}^{s} \simeq 1 \times (1.1 \times 10^3)^{\text{s}} \simeq (0.3 \div 4.5) \times 10^3,$$

where V_{eff} = 0.87 from numerical simulations, and $G_{\text{av}} \simeq 1114$ from Eq. (*1*).

Radiance enhancement factor. The radiance measures the intensity per unit solid angle and provides an estimate of the photon flux concentration, which is large when the photon flux has small divergence angle as in laser beams. The UCPL edge emission had a lateral size $< 100\ \mu$m. In the far-field where the optical fiber collector is placed, the beam did not extend farther than 200 μm. Thus, the solid angle of the radiation was below $\Omega_1 = \frac{\pi 0.1^2 \text{m}m^2}{5^2 \text{m}m^2} = 1.2$ msrad. The radiance enhancement, given as R_{EF}, is given by the ratio between the radiance R_1 of the UCPL edge beam and the radiance R_2 of the isotropic

UCPL bulk emission, which covers the full solid angle Ω_{sphere}, which is readily given by

$$R_{EF} = \frac{R_1}{R_2} = \frac{\Omega_{sphere}}{\Omega_1} \frac{(I'_a/\eta_1)}{(I_b/\eta_2)} \frac{\Delta t_b}{\Delta t'_a} \frac{P_b}{P'_a} \frac{N_b}{N_a} = \frac{\Omega_{sphere}}{\Omega_1} \times EF_{exp} \simeq 3.8 \times 10^8. \tag{7.5}$$

8 Additional FDTD simulations

A single dipole source was used to compute the isofrequency map using the *Z*-transform of the local optical field retrieved within the finite structure domain with the 3D full-field monitor. The intensity of the *Z*-transform determines the strength of radiation in the momentum space and better represents the radiation properties associated with the PCNS. To validate the results found with this approach, we first simulated a literature case discussed in ref. [5], i.e., supercollimation (selfcollimation) due to flat-band dispersion in the momentum space, which is shown **in Supplementary Fig. 13.**

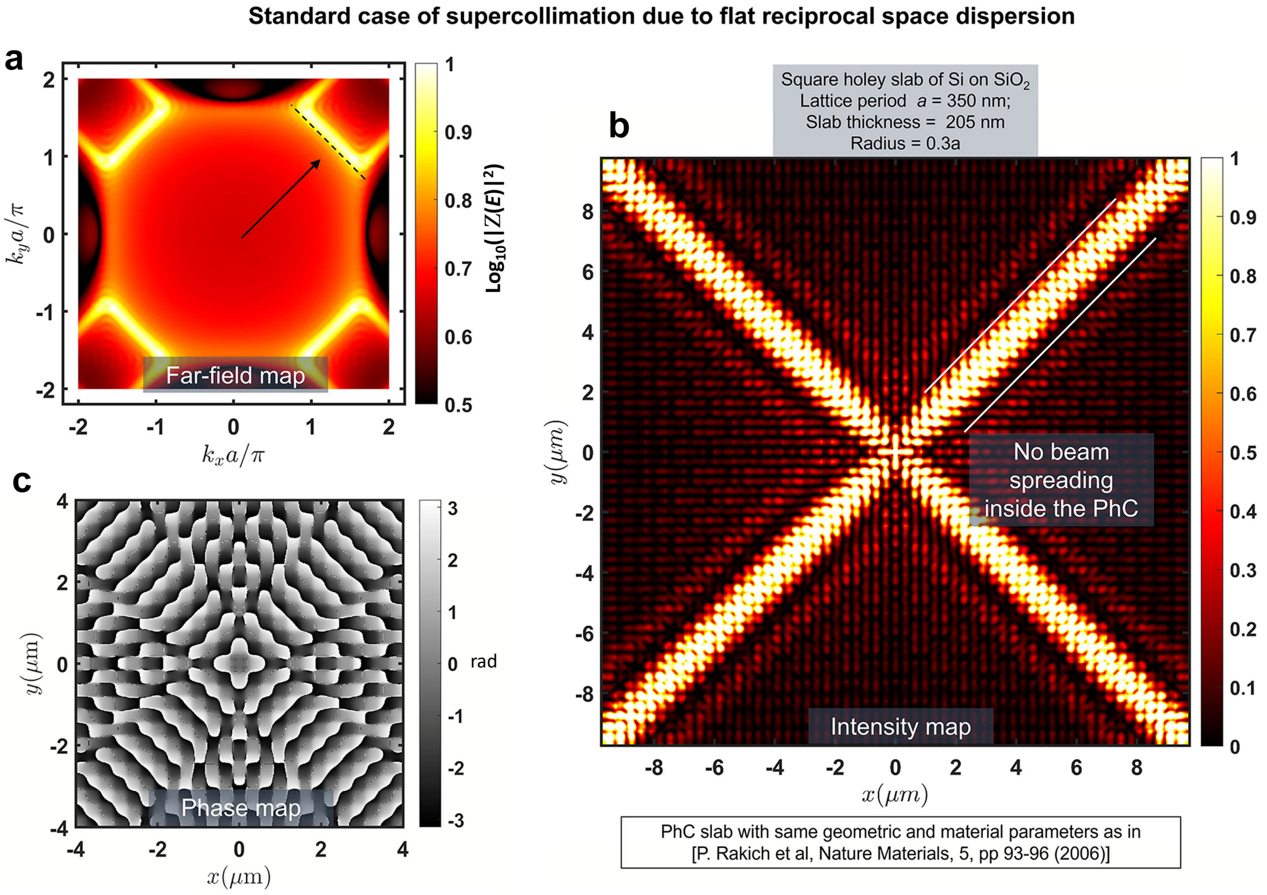

Supplementary Fig. 13 | a, Isofrequency far-field intensity map in the momentum space showing flat dispersion, in agreement with **b,** near-field intensity map showing self-collimation. **c,** Associated phase map.

9 UCPL emission scaling with pattern size

For each sample, we performed 30 acquisitions by moving the input beam along the side edge (**Supplementary Fig. 14**). It is important to note that the structure has a side length of 0.1 mm, consisting of 190 unit cells per side. The decrease in the signal is primarily attributed to the reduced quality factor of the resonator. However, slight modification in the band dispersion due to the size of the structure can also contribute to signal variations.

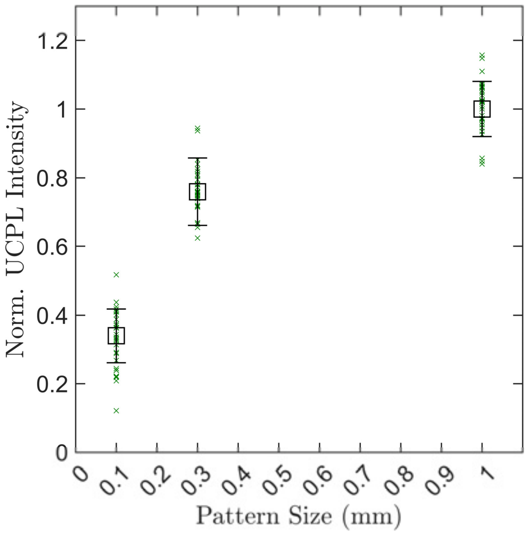

Supplementary Fig. 14 | Scaling of the intensity with the size of the pattern.

References

[1] H. Friedrich, D. Wintgen. Interfering resonances and bound states in the continuum. *Phys. Rev. A* **32**, 3231 (1985).

[2] N. Bernhardt, *et al.* Quasi-BIC Resonant Enhancement of Second-Harmonic Generation in WS_2 Monolayers. *Nano Lett.* **20**, 5309 (2020).

[3] S. Romano, *et al.* Ultrasensitive surface refractive index imaging based on quasi-bound states in the continuum. *ACS Nano* **14**, 15417 (2020).

[4] Kodigala, A., *et al.* Lasing action from photonic bound states in continuum. *Nature* **541**, 196-199 (2017).

[5] Rakich, Peter T., *et al.* Achieving centimetre-scale supercollimation in a large-area two-dimensional photonic crystal. *Nature Mater.* **5,** 93-96 (2006).
